# Novel radiation and targeted therapy combinations for improving rectal cancer outcomes

**DOI:** 10.1017/erm.2024.15

**Published:** 2024-04-16

**Authors:** Kathryn Pennel, Louise Dutton, Lydia Melissourgou-Syka, Campbell Roxburgh, Joanna Birch, Joanne Edwards

**Affiliations:** 1 School of Cancer Sciences, Wolfson Wohl Cancer Research Centre, University of Glasgow, Glasgow, G61 1BD, UK; 2 CRUK Scotland Institute, Glasgow, G611BD, UK; 3 Academic Unit of Surgery, Glasgow Royal Infirmary, University of Glasgow, Glasgow, G4 0SF, UK

**Keywords:** combination therapy, hallmarks of cancer, radiation, radio-sensitisation, rectal cancer, synergy, targeted therapy

## Abstract

Neoadjuvant radiotherapy (RT) is commonly used as standard treatment for rectal cancer. However, response rates are variable and survival outcomes remain poor, highlighting the need to develop new therapeutic strategies. Research is focused on identifying novel methods for sensitising rectal tumours to RT to enhance responses and improve patient outcomes. This can be achieved through harnessing tumour promoting effects of radiation or preventing development of radio-resistance in cancer cells. Many of the approaches being investigated involve targeting the recently published new dimensions of cancer hallmarks. This review article will discuss key radiation and targeted therapy combination strategies being investigated in the rectal cancer setting, with a focus on exploitation of mechanisms which target the hallmarks of cancer.

## Introduction

Rectal cancer is a leading cause of worldwide cancer-related mortality. Five-year survival is approximately 60%, dropping to <20% in patients diagnosed with metastatic disease (Ref. 1). Existing therapeutics for rectal cancer include chemoradiotherapy (CRT) followed by surgical resection. Analogous to colorectal cancer (CRC) in general, the biology of tumours arising within the rectum is heterogenous, with distinct differences in molecular oncogenesis, pathology, treatment options and responses. Accumulating evidence from the literature suggests specific targeted therapeutics may synergise with current treatments, particularly radiotherapy (RT). The mechanisms underlying these effects are associated with reversing/dampening acquired radio-resistance and/or promoting the anti-tumour effects of RT. This review aims to discuss recent advancements in the identification of therapeutically promising radiation: targeted therapy drug combinations for rectal cancer with a focus on the new dimension hallmarks of cancer.

### Conventional treatment of rectal cancer

The mainstay of rectal cancer treatment is surgical resection. This is usually preceded by neoadjuvant chemotherapy and/or RT. The rectum presents a difficult site for surgery, surrounded by vital organs, arteries and the pelvic bone; therefore surgery is considered more complex than for colon cancer. Consequently, full removal of the tumour is difficult; therefore, to improve outcome after surgery or even avoid the need for a more invasive surgical procedure, neoadjuvant RT is increasingly used in the clinic to gain local control and improve tumour margins to aid resection. The current gold standard surgical treatment for rectal cancer is total mesorectal excision (Ref. 2). Administration of CRT prior to surgery has been observed to reduce the incidence of local recurrence more than surgery alone (Ref. 3). A subgroup of patients obtains a pathological complete response (pCR) to the neoadjuvant therapy alone, and this has resulted in a move towards organ preservation (Ref. 3). In a recent phase II trial of locally advanced rectal cancer (LARC) (*n* = 324), total neoadjuvant therapy alone yielded pCR in 50% of patients and no difference in survival outcomes was observed between these patients who had no surgery versus historical control patients who received standard of care CRT followed by total mesorectal excision (Ref. 4). There is a hypothesis that optimisation of neoadjuvant treatment, potentially through combining RT with targeted therapies, may induce pCR in a larger proportion of patients thus reducing the frequency of invasive total mesorectal excision surgery.

RT describes the delivery of ionising radiation to a target volume, which for rectal cancer includes the tumour, mesorectum and pelvic lymph nodes (Ref. 5). The use of RT to treat rectal cancer has evolved over time (Ref. 6). It was initially adopted to manage local recurrences after surgery but is now utilised in the neoadjuvant setting. It can prevent local recurrences through ablation of micro-metastases within the pelvis. In terms of dosing there are now two commonly accepted regimes, long course and short course neoadjuvant RT. Long-course radiotherapy (LCRT) involves fractioned delivery of 1.8–2 Gy per day for 5 days out of 7 up to a total doses of 45–50 Gy. Surgical resection is then performed after 6 weeks (Ref. 5). Short-course radiotherapy (SCRT) involves hypo-fractionated doses of approximately 5 Gy per day for 5 days to reach 25 Gy with surgical intervention around 7 days after RT course (Ref. 5).

The selection of optimal RT treatment regime for rectal cancer patients varies worldwide and there are no specific guidelines for administration of SCRT over LCRT or vice versa. Ameta-analysis of eight studies comparing LCRT and SCRT carried out by Liscu *et al*. (Ref. 7) identified no significant difference in regards to severe, acute or late toxicities when using SCRT or LCRT schemes in neoadjuvant treatment of LARC. However, other studies note a favourable safety profile for SCRT supporting the concept that SCRT may be beneficial for more vulnerable patients such as the elderly or patients with poor performance status with comorbidities. This is because of the shorter treatment length and more mild acute side effects increasing the adherence to the regime without interruptions. In addition, patients who receive SCRT are less likely to require administration of chemotherapy alongside RT compared with LCRT (Ref. 8). There are additional benefits including higher adherence to the regime and reduced requirement for resources and costs associated with SCRT regimes (Ref. 8).

The current standard CRT regimes only achieve pCR in ~15% of cases. Although this is only a small proportion of patients, this subgroup may be able to avoid invasive surgery (Ref. 9). Therefore, if this group of patients could be expanded it would have huge clinical benefit. Identification of suitable targeted therapies which synergise with RT and prevent radio-resistance aims to increase the number of patients who obtain a pCR and therefore improve clinical outcomes.

### Preclinical models

The use of mouse models to test RT and targeted therapy combinations in the preclinical setting offers a fast and reliable way to explore the mechanisms of tumour response to the said treatments, yet the absence of anatomically relevant mouse models makes the clinical translation of preclinical findings challenging (Ref. 10). A number of different cancer-modelling techniques have been developed in mice over the years with varying degrees of complexity and clinical relevance. These models have been thoroughly described previously (Ref. 10), yet it is worth reviewing a few important characteristics of the animal models that can be employed to test combinations of RT and targeted therapies.

Subcutaneous xenograft mouse models developed through the injection of cell lines or tumour organoids constitute one platform that significantly reduces the latency of tumour growth and allows an easy and reliable method of measuring tumour response (Ref. 10). However, studying the effects of the tumour in its innate microenvironment becomes impossible. Genetically engineered mouse models (GEMMs) that were developed in the early 1990s have been instrumental in advancing our knowledge and understanding of tumour initiation and progression through different mutational phenotypes (Ref. 11). Technologies such as CRISPR/Cas-9 genome editing and the Cre-Lox recombinase have equipped the research community with mouse models that closely resemble colorectal adenocarcinomas, albeit at a high cost, long latency and limited ability to model metastatic disease (Ref. 10). A significant addition to the preclinical models' armamentarium was the development of transplant models, offering a high-throughput platform to test tumour response in an orthotopic setting. Surgically transplanted cell lines allow the study of early or invasive disease (Refs 12, 13), usually at the cost of a dysfunctional immune system through the use of immunocompromised mice to allow engraftment and the lack of relevant CRC histology. Other techniques such as chemical disruption of the colonic or rectal mucosa to model colitis-induced CRC is a reliable method to reflect some clinical scenarios (Refs 14, 15); however, this inflammation-inducing technique is often not of clinical relevance and it alters the tumour microenvironment (TME) in a way which may influence tumour response to treatment.

Recently, the development of colonoscopy-guided injection of organoids from GEMM-derived tumours into the colonic or rectal submucosa of immunocompetent mice offers a clinically relevant, fast and histologically reflective CRC modelling technique that is representative of the premetastatic CRC setting (Ref. 16). Such a model is particularly relevant in RT-immunotherapy (IO) studies, where the presence of a functional immune system is essential and the tumour response can be quantified in the original anatomical site. Outside of the animal model setting, patient-derived organoids (PDOs) can be used for personalised drug or therapy screening; however, this system completely ignores the role of the TME, which has shown to be a significant contributor to tumour response, or lack thereof (Refs 17, 18, 19). Considering the need for preserving the immune system, anatomical relevance, high-throughput and low latency, it is recommended that CRC preclinical testing platforms involve colonoscopy-guided orthotopic injection of tumour organoids in mouse models where possible.

The modelling of RT treatments in the preclinical setting has significantly advanced over the past few years through the development of the Small Animal Radiation Research Platform (SARRP; XStrahl) (Refs 20, 21). The SARRP allows the delivery of image-guided, fractionated RT to targeted anatomical areas, offering a reproducible environment, where normal tissue can be spared, inter-fractional variability reduced and side effects significantly limited (Ref. 22). The SARRP enables researchers to test clinically relevant fractionation regimes in combination with other targeted therapies in a fast and reproducible way. Although the description of preclinical studies that have employed the SARRP to test RT-targeted therapy combinations is outside the scope of this review, it is worth noting that RT displays dichotomising effects on the immune system and the microenvironment (Refs 23, 24). As such, it is essential that preclinical studies employ immunocompetent, orthotopic mouse models of rectal cancer.

### The new hallmarks of cancer

**Figure 1.**
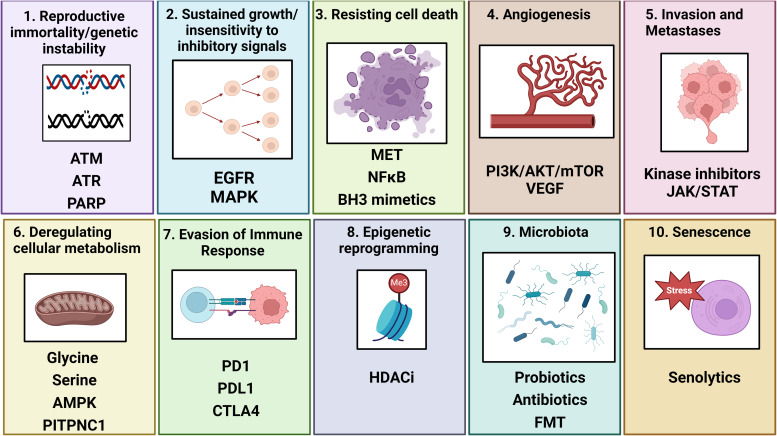
Hallmark mechanisms associated with resistance to radiation and associated potential targeted therapies to overcome this.

The hallmarks of cancer devised by Hanahan and Weinberg highlight the mechanisms tumour cells utilise to grow, survive and evade the host (Ref. 25). These hallmarks have identified many targets for therapeutic intervention across different tumour types and show the heterogenous and broad routes for cancer progression. The driving factors underlying tumour development and progression vary between tumour type and patients. The hallmarks of cancer were updated in 2022 and now consist of 12 discrete but linked mechanisms including genome instability and mutation, avoiding immune destruction, sustaining proliferative signalling, angiogenesis, invasion and metastases, deregulating cellular energetics, resisting cell death, evading growth suppressors, enabling replicative immortality, tumour promoting inflammation, senescence, polymorphic microbes, phenotypic plasticity and non-mutational epigenetic reprogramming (Ref. 26). Data suggest that targeting components of these hallmarks may improve the outcomes of cancer patients. There is also evidence that combining inhibitors which prevent or dampen these hallmarks could act synergistically with current therapeutic options including RT. Some of these potential combination strategies are highlighted in Figure 1.

## Hallmark 1: reproductive immortality and genetic instability

Radiation exerts its anti-tumour effects through damaging the genomic DNA of cancer cells. This damage includes single-strand breaks (SSBs), double-strand breaks (DSBs) and base and sugar damage (Ref. 27). To avoid destruction, tumour cells develop mechanisms to repair this damaged DNA (DNA damage response (DDR)) and can develop radio-resistance (Ref. 27). The main repair pathways include the homologous recombination (HR) pathway, non-homologous end joining (NHEJ) and alternative joining. Intermediate components of these pathways can be inhibited using targeted therapies. It is hypothesised that through targeting these pathways in combination with RT anti-tumour responses will be enhanced.

DDR inhibition combined with RT has been successful in the preclinical setting of rectal cancer. This includes use of small molecule ataxia telangiectasia mutated (ATM) kinase inhibitors, ataxia telangiectasia and Rad3-related (*ATR*) kinase inhibitors and enzyme poly-ADP ribose polymerase (PARP) inhibitors. For example, inhibition of ATM using AZ32 or KU-55933 in combination with photon and proton radiation significantly reduced viability of radio-resistant CRC PDOs (Ref. 28). Similarly, inhibition of ATR with AZD6738 with radiation increased the number and cytotoxic capacity of infiltrating CD8+ anti-tumour lymphocytes in an in vivo model of CRC (Ref. 29). The combination of AZD6738 and two fractions of 2 Gy in CT26 tumour-bearing mice caused a significant increase in the % of Ki67+ tumour-infiltrating CD8+ cells compared with irradiation (IR) alone at day 12 (*P* < 0.05) (Ref. 29). This was also observed amongst the splenic CD8+ population (*P* = 0.001) (Ref. 29). In a retrospective cohort of LARC patients who received neoadjuvant RT, low expression of both PARP1 (*P* = 0.001) and XRCC2 (*P* = 0.006) was associated with better overall survival (OS) (Ref. 30). In the same study the authors showed that in CRC cells lacking XRCC2, PARP inhibition with olaparib and RT reduced clonogenic capacity, increased DNA damage and senescence when compared with XRCC2 wild-type cells (Ref. 30). HCT116 and SW480 cells treated with olaparib and IR had significantly reduced colony formation compared with IR alone and this was potentiated in XRCC2 silenced cells (*P* < 0.05) (Ref. 30).

To date, there have been no clinical trials exploring the combination of any DDR inhibitor and RT in rectal cancer specifically. In a cohort of patients with advanced solid tumours (including CRC) a recent phase I clinical trial (NCI9938) investigating ATR inhibitor berzosertib with chemotherapeutic agent irinotecan was successful in demonstrating tolerability (Ref. 31). Recruitment is ongoing for phase 1/1b trial proposing the use of ATR inhibitor BAY1895344 in combination with FOLFIRI chemotherapy in gastrointestinal cancer (including CRC) patients (NCTO4535401). Another clinical trial in recruitment, PEMBROLA, is a phase 2 study designed to assess the combination of PARP inhibitor olaparib with a programmed death ligand-1 (PDL1) inhibitor in metastatic colorectal cancer (mCRC) HR-deficient patients (NCTO5201612). Future clinical studies to investigate DDR inhibitors in combination with RT should be undertaken given the tolerability of their use as single agents and the plethora of promising data from in vitro/in vivo work.

## Hallmark 2: sustained growth/insensitivity to inhibitory signals

It is well established that cancer cells can regulate their own growth through dysregulation of normal cell-cycle processes (Ref. 26). The production of excess growth factors within the tumour either by malignant cells themselves or through other cells in the TME potentiates this hyper-proliferative state. There are clinically approved drugs known to inhibit this uncontrolled cell division, such as cetuximab (anti-epidermal growth factor receptor (EGFR)), which has been used alongside chemotherapy for CRC patients over the past decade. There is now evidence that drugs such as cetuximab which target proliferation of the tumour cells may be able to enhance the effects of RT.

A retrospective study of 87 rectal cancer patients established a significant association between high expression of EGFR and reduced likelihood of pCR in pretreatment biopsies (*P* = 0.006) (Ref. 32). In this cohort 60% of patients had a biopsy which was determined EGFR positive, and detection of EGFR was independently associated with reduced disease-free survival (DFS) based on multivariate cox regression (radiation response (RR) = 2.88, 95% confidence interval (CI): 1.1–7.8, *P* = 0.036). In CRC cell lines, EGFR inhibitor gefitinib reduced clonogenic capacity and proliferation when used in combination with chemotherapeutic agent 5-fluorouracil (5-FU) and 3 × 2 Gy of radiation (Ref. 33). Similarly, another study showed a synergistic effect of cetuximab with radiation through inhibition of tumour cell growth in three CRC cell lines and in vivo using CRC nude mice bearing xenografts (Ref. 34). Combined treatment with a single fraction of 9 Gy and cetuximab (1 mg per mouse) significantly reduced tumour volume in HCT8, LoVo and WiDr xenografts (*P* < 0.05) compared with untreated controls (Ref. 34).

In terms of clinical trials, addition of cetuximab into CRT regimes has proven tolerable; however, limited effects on clinical outcomes have been observed in a phase 2 study (Ref. 35). Several other trials at phase I/II have yielded similarly disappointing results in terms of improving rectal cancer patient outcomes (Ref. 36). In another study pCR was only achieved in up to 20% of patients (Ref. 37). In a phase 2 study investigating therapeutic benefit of panitumumab (anti-EGF) combined with RT in *KRAS* mutant LARC (*n* = 19), the regime was well tolerated but no patients observed pCR (Ref. 38). The mechanisms underlying this are poorly understood and should be investigated to inform design of future novel combinations which target sustained proliferative signalling in tumour cells.

## Hallmark 3: resisting cell death

Resistance to cell death signals is a central characteristic of tumour cells. There are 13 different types of programmed cell deaths which tumour cells can develop resistance to; however, the most well established is apoptosis (Ref. 39). One of the best recognised mechanisms of radio-resistance involves tumour cells becoming resistant to apoptotic signals. Inhibiting these anti-apoptotic signals may be achieved by targeting aberrant cellular signalling pathways such as c-MET or nuclear factor kappa B (NF*κ*B). There is now a mounting body of evidence demonstrating that expression of anti-apoptotic proteins in tumour cells may be influenced by RT, and inhibiting this resistance to cell death may enhance response to CRT. For example, anti-apoptotic protein X-linked inhibitor of apoptosis protein expression has been observed to be increased in serially collected rectal cancer biopsies (*n* = 29) when measured by western blot (Ref. 40). This increase in the level of expression correlated with resistance to treatment (Ref. 40).

Similarly, an upregulation of anti-apoptotic proteins such as B Cell lymphoma 2 (Bcl-2) and myeloid cell leukemia sequence 1 (Mcl-1) has also been observed following radiation in multiple cancer types (Ref. 41). These proteins can be targeted using BH3 mimetics, which have shown efficacy when tested as monotherapy in multiple CRC cell lines (Ref. 42). There is limited evidence in terms of BH3 mimetics and RT combinations in rectal cancer specifically; however, inhibition of Mcl-1 radio-sensitised melanoma cell lines (Ref. 43). In breast cancer, inhibition of Bcl-2 using ABT-737 in cell lines synergised with RT treatment via regulation of Mcl-1 (Ref. 44). In this study, there was 39.7% surviving clones in ABT-737 as monotherapy versus only 11.2% in ABT-737 in combination with 4 Gy (*P* < 0.01). ABT-737 also enhanced response to radiation when tested using in vitro and in vivo models of lung cancer (Ref. 45). These data suggest that targeting anti-apoptotic proteins should be investigated preclinically in the rectal cancer setting.

Treatment of CRC cell lines with curcumin which inhibits NF*κ*B signalling potentiated the response to 5-FU and when tested in combination with RT in CRC xenografts a reduction in tumour growth was observed (Refs 46, 47). In the xenograft study the proliferation index of tumours treated with radiation and curcumin combination was significantly lower than that in the RT-alone arm (*P* < 0.001). An inhibitor of 26S proteasome, bortezomib, also hits I*κ*B in vitro which results in inhibition of canonical NF*κ*B signal transduction. In a phase I trial in rectal cancer patients (*n* = 9), when bortezomib was combined with RT drug-associated toxicity terminated the study early. Further work to characterise the effect of non-canonical NF*κ*B signalling inhibition in combination with radiation using in vitro/in vivo models should be performed to avoid toxicities associated with canonical signalling.

The multi-kinase inhibitor sorafenib synergised with radiation in vitro and in vivo models of rectal cancer via its suppression of NF*κ*B signalling (Ref. 48). This links with resistance to cell death because of the prominent role that NF*κ*B plays in promoting apoptosis resistance in CRC (Ref. 49). In this study a HT29/*tk-luc* tumour-bearing nonobese diabetic (NOD)/severe combined immunodeficient (SCID) mouse model was employed, and 35 days after initiation the control arm tumour volume was 20× the size of the initial tumour (Ref. 48). The tumours treated with IR alone (1 × 12 Gy) were 5× the initial volume and tumours treated with a combination of a single 12 Gy fraction and sorafenib were only 1.5× greater than the initial tumour volume (Ref. 48). The tumour volume of the IR-alone arm was significantly lower than the combination treatment (*P* < 0.005) (Ref. 48).

Utilisation of multi-kinase inhibitor sorafenib in clinical trials has yielded disappointing results to date. In phase I/II clinical trials (NCT00869570) the drug was well tolerated in combination with RT and capecitabine, but outcome measures were not conclusive. In the context of *KRAS* mutant rectal cancers results have been more promising. In a phase I–II trial (*n* = 54) sorafenib combined with 25 fractions of 45 Gy administered of 5 weeks resulted in a pCR of 60% and limited toxicity (Ref. 50).

In a multicentre study, high expression of c-MET in biopsies (*n* = 81) was found to correlate with reduced responsiveness to RT in rectal cancer (Ref. 51). A novel c-MET inhibitor PHA665752 synergised with RT in vivo through induction of apoptosis and reduction of hypoxia inducible factors (HIF)-1*α* expression in cell line/murine xenografts (Ref. 52). Similarly, novel antibodies targeting c-MET acted synergistically with RT in CRC cell lines and in murine xenograft models (Ref. 53). Data from clonogenic assays from this study showed that HT29 cells treated with a combination of 2 Gy and seeMet 12 compound (c-MET inhibitor) had significantly reduced colony formation than 2 Gy monotherapy (*P* < 0.001) (Ref. 53). This was potentiated when the radiation dose was escalated to 4 Gy (*P* < 0.001) (Ref. 53). These data are corroborated by findings from a study which found c-MET inhibitor crizotinib radio-sensitised a panel of KRAS mutant CRC cell lines (Ref. 54).

## Hallmark 4: angiogenesis

In order for a tumour to be supplied with sufficient nutrients and oxygen there is continual growth of vasculature termed angiogenesis (Ref. 25). In normal homoeostatic processes angiogenesis occurs transiently; however, in the cancer setting it can become permanently switched on. This is associated with poor prognosis and therefore inhibitors of molecules involved in vessel formation are currently utilised for CRC in combination with chemotherapy. For example, bevacizumab is a biologic which inhibits tumour angiogenesis through blockade of vascular endothelial growth factor receptor (VEGFR). Bevacizumab is currently used clinically in the metastatic CRC setting in combination with adjuvant chemotherapy (Ref. 55). There is now growing evidence that targeting angiogenesis in combination with RT may hold clinical promise.

Evidence from preclinical studies of other solid tumour types has implicated a promising role of vascular endothelial growth factor (VEGF) inhibition as a synergistic therapy for radiation. For example, vascular endothelial growth factor receptor 2 inhibition in mouse xenografts enhanced the effect to radiation in squamous cell carcinoma (Ref. 56). Similarly in models of murine lung cancer models treatment with VEGFA inhibitor famitinib showed synergistic effects with radiation administration (Ref. 57). Evidence is more limited for in vivo models of rectal cancer specifically. In a xenograft model of CRC, VEGFR inhibitor cediranib synergistically enhanced the effect of radiation via reducing hypoxia and vascularisation of the TME (Ref. 58). The hypoxia rate of subcutaneous tumours was measured by pimonidazole staining and this showed a significant decrease in hypoxia in the cediranib RT combination arm versus RT alone (*P* < 0.005) (Ref. 58). Unlike VEGFR, inhibition of FGFR in vivo failed to show any radio-sensitisation effects (Ref. 59).

Several clinical trials have now explored the potential for inhibiting angiogenesis to enhance the effects of neoadjuvant CRT for rectal cancers. A phase 2 clinical trial which incorporated VEGF inhibitor bevacizumab with capecitabine and RT in LARC yielded disappointing results with no tangible effects of combined therapy on pCR, relapse rates, DFS or OS (Ref. 60). The BACCHUS trial (NCT01650428) was a randomised phase 2 study of bevacizumab and SCRT with FOLFOXIRI in LARC which proven tolerability of the combination treatment, however failed to reach its endpoints (Ref. 61).

In addition to VEGF, mammalian target of rapamycin (mTOR) inhibitors have also been investigated to combat angiogenesis in rectal cancer. When PDOs which had shown radio-resistance were sequenced, gene set enrichment revealed upregulation of genes associated with epithelial to mesenchymal transition (EMT) and PI3K/protein kinase B (AKT)/mTOR. Radio-resistant HCT116 cell lines and PDOs’ dual inhibition of PI3K/mTOR were sensitised to radiation (Ref. 62). In two PDO lines apitolisib (PI3K/mTOR inhibitor) combined with 5 fractions of 5 Gy significantly reduced viability when compared with drug alone (*P* < 0.001) (Ref. 62). Similarly, dactolisib (PI3K/mTOR inhibitor) and 5 fractions of 5 Gy in the same two PDO lines significantly reduced viability compared with drug alone (*P* = 0.015, *P* = 0.002) (Ref. 62). An additional study observed that protein expression of phosphorylated Akt at serine 473 was elevated in rectal biopsies of patients who had poor response to CRT (*n* = 50) (Ref. 63). In the same study, Akt inhibitor MK0026 reduced the clonogenic potential of SW480 radio-resistant CRC cell lines when assessed in vitro and in vivo as murine xenografts (Ref. 63). In clonogenic assays the survival fraction of SW480 radio-resistant cells was reduced by >50% when treated with a combination of 2 Gy and MK0026 compared with control cells (Ref. 63). A phase I–II trial demonstrated tolerability of mTOR inhibitor rapamycin in combination with SCRT in primary resectable rectal cancer; however, no effect on tumour regression was detected despite alteration to metabolism (Ref. 64). This may be because of no predictive biomarkers being employed.

## Hallmark 5: invasion and metastases

The hallmark of invasion and metastases is strongly associated with cancer progression and outcome and is a leading cause of morbidity and mortality in cancer patients (Ref. 25). Data now suggest RT itself can induce processes involved in invasion and metastasis, making this a key hallmark for investigation in the context of enhancing response to RT (Refs 65, 66). The process of invasion begins with changes to the tumour cells at the primary site of origin including down-regulation of surface-bound adhesion molecule E-cadherin (Ref. 67). This initiates a cascade of events termed epithelial to mesenchymal transition (EMT) which regulates the process of invasion and metastases (Ref. 67). When tumour cells go through EMT local invasion occurs whereby individual or clusters of tumour buds intravasate into the local lymphatic and vascular systems. These cells can form micro metastases locally or migrate to distant sites (liver, lung) where they can adapt to unfamiliar environments and colonise as metastases. The process of EMT is underpinned by upregulation of specific transcription factors (TFs), receptors and signal crosstalk with cancer-associated stroma. Targeting EMT directly has proven challenging unless tumours are detected and treated before the intravasation processes have started. Inhibiting dysregulated signalling pathways which promote EMT has been better characterised, and there is now evidence that this could be used to enhance the effects of RT.

Targeting transcription factors or intermediate signalling components related to the EMT cascade itself is relatively understudied. To date research has focused on targeting more indirect mechanisms of EMT blockade including through inhibition of Janus kinase (JAK)/signal transducer and activator of transcription (STAT) signalling and multi-kinase inhibitors. Initial studies have observed that phosphorylation of STAT3 is induced by radiation dose dependently in cell lines (Ref. 68). In mouse rectal cancer xenografts STAT3 inhibitor napabucasin in combination with CRT significantly reduced tumour growth and angiogenesis (Ref. 69). HCT116 xenografts treated with a combination of 2 × 4 Gy and napabucasin showed significantly reduced tumour volume (mm^3^) compared with the IR-alone treatment group (*P* < 0.005) at 15 days post-subcutaneous injection (Ref. 69). In vitro napabucasin also increased the gamma-H2AX expression to a greater extent that CRT treatment alone (Ref. 69). Similarly in subcutaneous xenografts, inhibition of STAT3 using Stattic enhanced the anti-tumour action of RT (Ref. 70). The mechanisms underlying this enhanced effect to RT with STAT3 inhibition are not solely related to EMT. There is evidence that STAT3 can actively modulate ATM and ATR DDR pathways through transcriptional regulation of MDC1. Knockdown of STAT3 results in reduced capacity to repair DNA in MEF cell lines (Ref. 71). In HCT116 CRC cell lines, inducing DNA damage increased interleukin (IL)-6 secretion peaking at 48 h after stimulation (*P* < 0.001) and activation of JAK1 and STAT3 peaking at 72 h. Inhibiting the pathway in these cells induced senescence and halted cellular growth (Ref. 72). IL-6 has been observed to initiate binding of STAT3 to Toll-like receptor 9 in myeloid cell populations in vivo mouse models of rectal cancer. This coupling results in increased angiogenesis and tumour growth, and thus inhibition of STAT3 increased the radio-sensitivity (Ref. 73). Another mechanism through which STAT3 facilitates radio-resistance is by activation of NOTCH signalling (Ref. 74). This makes these inhibitors an attractive route for radio-sensitisation because of their pleiotropic effects. There have been no clinical trials commenced to date for combined therapy with RT in rectal cancer. STAT3 inhibitor, TTI-101, is undergoing phase 1 clinical trial (NCT03195699) currently for advanced stage CRC. A recent phase II study of pacritinib (JAK inhibitor) for refractory CRC was terminated early because of toxicity (NCT02277093).

Silencing of doublecortin-like kinase 1 in HCT116 CRC cell lines significantly reduced colony formation, and increased apoptosis and cycle arrest compared with wild type following treatment with 4 and 6 Gy irradiation (Ref. 75). The mechanisms underlying this may relate to EMT processes. After IR siDCKL1 HCT116 cells had significantly reduced expression of EMT-related genes compared with control cells (*P* < 0.01) (Ref. 75). A recent phase II basket trial (NCT04346381) investigating the utility of combining programmed death protein-1 (PD1) inhibitor camrelizumab with receptor tyrosine kinase inhibitor famitinib reported particularly promising results amongst the rectal cancer (RC) group (*n* = 30, stage IV) and good tolerability (Ref. 76). There are a plethora of alternative kinase inhibitors which could be assessed for their clinical utility in combination with RT.

## Hallmark 6: deregulating cellular energetics

Radiation exerts its effects on cancer cell DNA either in a direct manner via DSBs or SSBs, or through the radiolysis of water and other intracellular molecules, resulting in release of reactive oxygen species (ROS), leading to oxidative stress (Ref. 77). This induces multiple processes harmful to cellular viability, including lipid peroxidation, protein oxidation and DNA damage. Lesions induced by these processes can remain unrepaired, resulting in genomic instability and subsequent cell death (Ref. 78). Therefore, success of RT is somewhat attributed to the superior ability of normal cells to repair this DNA damage compared with cancer cells. DNA repair is an energy-demanding process – glucose and amino acid metabolism are required to generate nucleotides for this and therefore targeting radio-resistance through metabolism poses a promising therapeutic approach.

The alteration of metabolism is a prominent hallmark of cancer which is linked to sustaining the highly proliferative nature of tumour cells (Ref. 26). Cancer cells can acquire radio-resistance through rewiring their metabolic pathways, developing mechanisms to enhance their DNA damage repair and antioxidant defences, thereby mitigating the cytotoxic effects of ionising radiation. This is evidenced in rectal cancer by Buckley *et al*. (Ref. 79), whereby the inherently radio-resistant rectal cell line SW837 displayed enhanced DNA damage repair compared with the radio-sensitive HCT116 cell line. Transcriptomic profiling of the two lines revealed significant differences in metabolic pathway activation, such as oxidative phosphorylation having 30% of associated genes overexpressed in the radio-resistant SW837 cells compared with the radio-sensitive HCT116s, suggesting altered metabolism may play a role in promoting radio-resistance. The importance of metabolism is further demonstrated by the analysis of the metabolome of pretreatment sera from RC patients where significant alterations between patients with a poor response to neoadjuvant CRT and poorer survival were observed. This indicates the potential for metabolites to be used as circulating biomarkers to predict response in RC patients. However, the challenges of targeting metabolism in combination with RT was demonstrated by a differential response to the combination of RT and the glycolysis inhibitor, 2-deoxy-d-glucose (2-DG) in the two lines tested, where only the radio-sensitive HCT116 responded.

Along with the profound effects on DDR pathways, metabolic reprogramming affects additional mechanisms, contributing to cancer progression, metastasis and treatment resistance. In varying cancer types, including RC, cancer cells have been shown to rewire metabolism to aid their rapid cell proliferation, resulting in abnormal glycolysis and lipid synthesis (Ref. 80). In addition, malignant CRC tumours are capable of metabolic rewiring to activate components of the TME, altering signalling pathways and metabolites (Ref. 81).

Metabolic markers which predict pCR or poor responses to neoadjuvant chemoradiotherapy (NCRT) have been identified. RNASeq data from a study of 381 patients revealed an association between metabolism-related pathways and poor outcomes (Ref. 82). These pathways from the hallmark gene sets included protein secretion, glycolysis, xenobiotic metabolism and haem metabolism (Ref. 82). These data highlight a general role for metabolism is dictating response to RT in RC. A more specific study in 2021 established pretreatment circulating levels of metabolic marker paraoxonase-1 was predictive of good response to neoadjuvant CRT in rectal cancer patients (*n* = 32) (Ref. 83). Conversely, low levels of systemic succinate were indicative or disease relapse in a cohort of 48 rectal cancer patients receiving NCRT (Ref. 83). This highlights the importance of the metabolome in terms of predicting response to RT, and the potential for developing therapeutic targets to alter metabolism to evoke better response to RT in patients with rectal cancer.

Another metabolic marker implicated in the context of rectal cancer response to RT, adenosine monophosphate activated kinase (AMPK), has been detected at higher levels in rectal cancer tumours which don't respond to radiation (*n* = 5) (Ref. 84). AMPK plays an important role in metabolic reprogramming and cell growth. Using a preclinical inhibitor of AMPK or disrupting expression via RNAi results in a synergistic effect of radiation when tested using in vitro models of CRC (Ref. 84). Other studies have targeted mitochondrial respiration to overcome radio-resistance using biguanides and atovaquone. These drugs have been demonstrated to reduce hypoxia within the TME thereby promoting increased DNA damage.

There have been several other targeted therapeutic approaches which alter metabolic activity. Research is also focused on a more general approach of altering the presence of certain nutrients, to avoid metabolic pathway redundancy often observed with more targeted approaches. For example, in CRC cell lines restriction of serine and glycine induced radio-sensitising effects (Ref. 85). HCT116 cells cultured in the absence of serine and glycine and treated with 2 Gy IR had significantly reduced colony formation compared with cells grown with serine and glycine present (*P* < 0.005) (Ref. 85). This was also observed for 4T1 (*P* < 0.0005), DLD1 (*P* < 0.00005) and EO771 (*P* < 0.0005) cells (Ref. 85). This is supported by data from a cohort of 54 advanced rectal cancer patients showing that high pretreatment glycine concentrations within the tumour are independently associated with reduced progression-free survival (Ref. 86). Phosphatidylinositol transfer protein, cytoplasmic 1 (PITPNC1) is another metabolism-associated therapeutic target potentially identified for radio-sensitisation of rectal cancers. High gene expression of *PITPNC1* predicts radio-resistance in rectal cancer tissue (*n* = 16) and cell lines (*n* = 2) through inhibition of ROS production (Ref. 87), whereas genetic ablation of the gene restored ROS generation (Ref. 87).

Recent studies have implicated cancer-associated fibroblasts (CAFs) as modulators of tumour cell metabolism in the context of RT. In vitro, CAFs subjected to radiation promote survival of CRC cell lines (Ref. 88). This was because of a rewiring of the CRC cell metabolism to higher glutamine consumption in an IGFR-dependent manner. Subsequent investigation in orthotopic models demonstrated a reduced formation of metastases when IGF1 was neutralised following RT (Ref. 88). These results corroborate data from a murine glioma study which established a radioprotective role for IGFR1 ex vivo and showed blockade of IGFR1 increased the sensitivity of glioma stem cells to radiation (Ref. 89).

Data from these preclinical studies suggest that deregulation of cellular energetics represented a promising hallmark for targeting to enhance response to RT. Further investigation into the metabolic reprogramming in radio-resistant RC may allow for prediction of neoadjuvant CRT response and the development of novel therapeutics.

## Hallmark 7: avoiding immune destruction

Harnessing the effect of radiation on surrounding immune populations represents an exciting field. In terms of CRC more generally, IO has been utilised clinically in a subpopulation of patients with mismatch repair (MMR) deficient disease. Research has shown that the DNA damaging effects of RT causes release of damage-associated molecular patterns from the affected cancer cells (Ref. 90). In addition, neoantigens, proinflammatory cytokines and cytosolic DNA are released into the TME which orchestrates an immune priming effect on anti-tumour populations. This includes influx and proliferation of resident CD8+ cytotoxic T cells and recruitment of antigen presenting cells such as dendritic cells. RT has been shown to increase expression of major histocompatibility complex 1 on the surface of tumour cells enabling easier recognition by cytotoxic T cells (Ref. 91). However, there is also some evidence that radiation can increase the presence of FOXP3+ regulatory T cells enhancing immune suppression. Further research is required to fully characterise the immune response to RT, with inter-patient heterogeneity likely to highlight the need for biomarker identification.

Immune checkpoint inhibitors are perhaps the most well-studied targeted therapies in the setting of radio-sensitisation of rectal cancer. These are a group of protein expression on the surface of T cells which bind to specific cognate proteins expressed on the surface of host cells including antigen presenting cells. This interaction is required for T cell activation and recognition of self. In the cancer setting, tumour cells can express immune checkpoint molecules, which prevents activation of the T cells leading to immune evasion. In rectal cancer a key pair of immune checkpoint proteins includes PDL1 and PD1 (Ref. 92).

In the preclinical setting the combinations of PD1/PDL1 and RT have been well characterised (Ref. 93). This has culminated in the development of numerous clinical trials. Given the immunomodulatory effects of radiation, several clinical trials are underway investigating the combination of checkpoint inhibitors with either LCRT or SCRT for rectal cancer patients. In phase II trials the combination of SCRT and camrelizumab (anti-PD1) produced promising results in terms of pathological response with potentiation in MMR-proficient cases (clinical trial ID NCT04231552) (Ref. 94). A similar phase II trial using anti-PD1 agent toripalimab with 5 × 5 Gy SCRT is ongoing (NCT04518280). A study of pembrolizumab with CRT produced disappointing results, yielding no differences between control and treatment arms in terms of neoadjuvant rectal score or pathological responses, despite the combination being well-tolerated (Ref. 95). Inhibitors of the PD1 ligand, PDL1, such as durvalumab, are being investigated in the context of CRT in clinical trial PRIME-RT (Ref. 9).

In addition to PD1/PDL1 targeting other immune checkpoint molecules such as cytotoxic T-lymphocyte-associated protein 4 (CTLA-4) and T cell immunoreceptor with Ig and ITIM domains (TIGIT) in combination with RT represent exciting therapeutic approaches for rectal cancer. In a murine model of rectal cancer RT combined with CTLA-4 inhibition reduced formation of metastases. In the same study RT plus anti-CTLA-4/CD25 monoclonal antibody treatment reduced tumour growth, increased cytotoxic T cell infiltrates and increased overall survival (Ref. 96). In subcutaneous mouse models, expression of TIGIT was increased in response to administration of 3 × 8 Gy radiation (Ref. 97). When TIGIT and PDL1 were blocked in combination with RT pCR was 90% compared with 80% in mice treated with anti-PDL1 and radiation (Ref. 97). Recent clinical trials have investigated the combination of CTLA-4 inhibitors with PDL1 inhibitors and low dose fractionated or hypo-fractionated RT in stage 4 microsatellite stable rectal cancer patients (*n* = 18). Data were inconclusive and suggestive of high levels of toxicity (Ref. 98).

In addition to checkpoint blockade, oncolytic viruses have been investigated in clinical trials as a method to modulate the immune landscape on rectal cancer patients. Analysis of results from the phase 1 CEDAR trial (NCT03916510) which assessed a combination of oncolytic virus enadenotucirev, capecitabine and 50 Gy RT in 30 patients are ongoing (Ref. 99).

## Hallmark 8: epigenetic reprogramming

Epigenetics refers to stable heritable modifications to chromosomes without alteration to the DNA sequence. As advances in the understanding of epigenetic modifications are made, they become more closely linked to the initiation and progression of cancer, with key mechanisms including DNA methylation, histone acetylation and histone methylation, which are now accepted as a hallmark of cancer (Refs 26, 100). As these are reversible responses, targeting these mechanisms of tumour promotion and progression through reprogramming is of high significance and has the potential to synergise with existing therapeutics including RT. Importantly, as RT can cause genetic changes to the DNA of cells, it can also affect epigenetics, and some reports suggest that although RT induces cell death in the majority of tumour cells, it has the potential to induce radio-resistance via inducing more aggressive epigenetic phenotypes (Ref. 101). Despite this, current research has demonstrated that a number of epigenetic modifiers act as radio-sensitisers, including deacetylase (HDAC) inhibitors, DNA methyltransferase (DNMT) inhibitors, enhancer of zeste 2 polycombe repressive complex 2 subunit (EZH2) inhibitors and bromodomain and extra terminal domain (BET) inhibitors (Ref. 102), via destroying DNA-damage repair and cell cycle, in addition to increasing oxidative stress (Ref. 103).

DNA methylation is arguably the most understood aspect of epigenetics and contributes to a phenotype of CRC termed ‘CpG island methylator phenotype (CIMP)’ which occurs in around 20–30% of CRCs. The pathway comprises atypical DNA hypermethylation in CpG dinucleotide sequences situated within promoter regions of regulatory genes involved in cell cycle, apoptosis, DNA repair, angiogenesis and cell invasion and adhesion (Ref. 104). Loss of gene expression occurs in those harbouring promoter region CpG island hypermethylation, as it causes the chromatin to close and form a physical block, preventing transcription factor binding and as such resulting in transcriptional silencing (Refs 104, 105). RT may induce changes to methylation status and DNMT; however, this poses potential for both positive and negative effects (Ref. 106). Therefore, epigenetic status prior to, and post-RT, is of importance. For example, DNMT3B silencing has been shown to demethylate p53 and p21, restoring their functions and subsequently inducing cell-cycle arrest and apoptosis. However, RT has been shown to increase DNMT3B and therefore the methylation of p53 and p21, consequently promoting radiation-resistance in nasopharyngeal carcinoma (Ref. 107).

Histone modification is another key regulatory mechanism in epigenetics, involving histone methylation, acetylation, phosphorylation and ubiquitination and is acknowledged as an essential mechanism involved in RC initiation and progression. Specifically, histone acetylation has been implicated in regulating a variety of cellular functions, including differentiation, changes in chromatin structure and gene expression stability. Importantly, the capacity of cancer cells to repair radiation-induced DNA damage, thus preventing cell death, can be inhibited by HDACis, via the DNA damage signalling and repair pathways (Ref. 108). Therefore, one of the key targets for epigenetic reprogramming is HDACis, which have gained traction in clinical trials. To date these have only been studied preclinically in RC; however, this has yielded promising data. In co115 and KM20L2 rectal cancer cell lines, inhibition of HDAC using suberoylanilide hydroxamic acid (SAHA; currently licensed as vorinostat) or the benzamide MS-275 (both HDAC inhibitors in clinical development), reduced clonogenic potential when treated in combination with ionising radiation (Ref. 109). In HCT116 TP53 wild-type cells, HDAC inhibitor valproic acid reduced xenograft tumour growth after radiation (*P* < 0.05) (Ref. 110).

Overall, although epigenetic modifiers have demonstrated promise, they should be advanced in combination with RT with caution, as epigenetic status of the patient prior to and following RT is vital.

## Hallmark 9: microbiota

The microbiome in cancer patients is a double-edged sword, as cancer therapies can cause dysbiosis which then leads to resistance to therapeutic response (Ref. 111). Harnessing the power of manipulation of the microbiota to improve cancer patient outcomes is an area of ongoing research. Studies have demonstrated that RT causes dysbiosis (Ref. 112). This leads to disruption of the mucous layer and intestinal barrier and inflammation (Ref. 111). There are very limited mechanistic studies to date which explore the role of the microbiome in dictating response to RT, and that explore modulation of the microbiome to reduce resistance. Murine models to study host–microbe interactions are under development and will improve this area of research (Ref. 113). In a recent study, Dong *et al*. reported that the oral microbiota in CRC mouse models influences responses to RT and migration of a specific species (*Fusobacterium nucleatum*) to the tumour region reduces efficacy or RT (Ref. 114). In vitro studies have demonstrated that antibiotics such as cephalosporin can act as radio-sensitisers in CRC cell lines (Ref. 115).

The microbiotic landscape of a cohort of rectal cancer patients (*n* = 84) receiving neoadjuvant LCRT demonstrated clear differences in profiles in baseline stool samples between responders and non-responders. Species such as *Coriobacteriaceae* and *Fusobacterium* were enriched in the non-responder group (Ref. 116). A further study has confirmed that neoadjuvant RT impacts diversity of the microbiome through 16s sequencing of faecal samples from a larger cohort of 353 rectal cancer patients (Ref. 117). In terms of clinical interventions, a study of 917 patients with pelvic/abdominal cancers showed that administration of a probiotic supplement pre-RT reduced treatment-related side effects (Ref. 118). Patients who received probiotic supplement (*n* = 490) were significantly less likely to develop RT-induced diarrhoea than patients who received placebo treatment (*n* = 427) (RR = 0.55, 95% CI: 0.34–0.88, *P* = 0.01) (Ref. 118).

## Hallmark 10: senescence

Cellular senescence is defined by irreversible cell-cycle arrest as a response to both intrinsic and extrinsic stress stimuli including ageing, oxidative stress or DNA damage. In addition, it has become increasingly acknowledged that both chemotherapy and RT can also induce this state of irreversible proliferative arrest, termed ‘therapy-induced senescence’ (TIS) in both tumour and stromal cells (Ref. 119). Ionising radiation induces senescence via the induction of the DDR, NHEJ and HR, pathways heavily involved in the repair of DSBs and are potent initiators of cellular senescence. Notably, CRC patients with sporadic senescent cells detected prior to treatment had an increased likelihood to TIS and subsequently improved response to adjuvant chemotherapy (Ref. 120). However, despite its previously established role as a tumour suppressive mechanism by inhibiting the proliferation of premalignant cells, growing evidence indicates that senescent cells can also promote tumour progression, as they stay metabolically active and secrete an array of factors including pro-inflammatory cytokines, chemokines, growth factors and matrix-remodelling proteases into the microenvironment in a process termed senescence-associated secretory phenotype (SASP) (Ref. 121). The downstream effects of SASP are dependent on cell type and vary between differing stages of cancer progression (Ref. 122). It has been demonstrated, however, that SASP can induce a more aggressive phenotype in non-senescent cells via paracrine signalling, as well as exerting detrimental effects on the surround tissue microenvironment. In vitro studies of two colon cancer cell lines and one rectal cancer line identified that senescent cancer cells were capable of consistently secreting multiple cytokines, including IL-8, and that these senescence-associated secretomes caused paracrine affects such as increased proliferation, invasiveness and induction of EMT. In addition, their investigation of clinical samples from neoadjuvant CRT patients identified that senescence and EMT co-occurred within cancer cell clusters (Ref. 123). In addition to the effect on cancer cells themselves, ionising radiation can also induce senescence and associated SASP in surrounding stromal cells, leading to tissue fibrosis, organ dysfunction and long-term inflammation (Ref. 124).

One hallmark of senescence is the change in chromatin structure, consequently altering gene expression. This can affect downstream processes such as apoptosis regulation, causing new vulnerabilities specific to these senescent cells, and therefore be specifically targeted. Senolytics are one of the agents investigated for the selective killing of senescent cells; however, the broad range of activity, safety and specificity from the current generation of senolytics provides obvious limitations, although preliminary data demonstrate potential in combination with RT. For example, normal tissue damage induced by RT was reduced by alvespimycin (17-DMAG), an HSP-90 inhibitor, without comprising RT effectiveness (Ref. 125).

Overall, the role of senescence in cancer remains complex, however was notably identified as an emerging hallmark of cancer. As such research investigating this phenomenon in the context of cancer has gained traction. Advances in the understanding of the molecular and cellular pathways involved in senescence will provide novel strategies to either enhance RT or reduce normal tissue damage induced by RT.

## Conclusions

There is an unmet need for the identification of novel therapeutic strategies to improve the clinical outcomes of rectal cancer patients. A vast body of research over the past decade has focused on mechanisms to exploit, enhance or improve the therapeutic benefit of RT in relevant solid tumour types. The review has highlighted evidence for potential benefit of combination treatment of targeted therapies which inhibit specific hallmarks of cancer with RT in rectal cancer.

**Figure 2.**
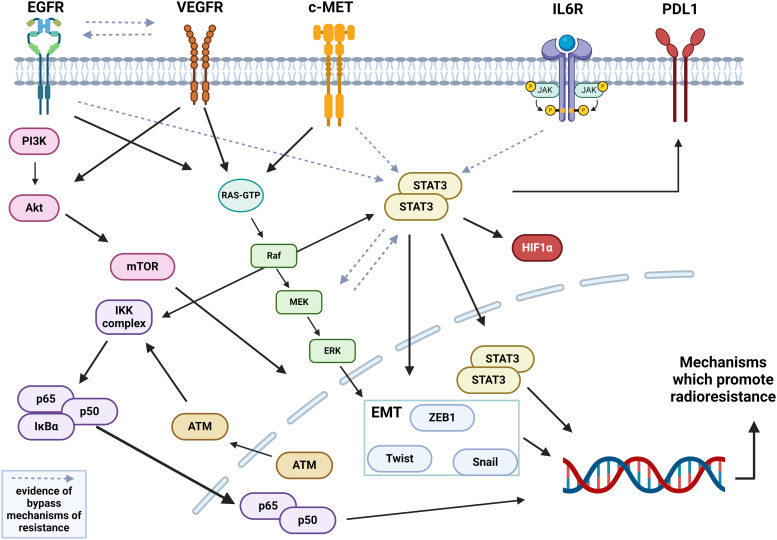
Cell signalling pathways collaborate to induce resistance to RT.

In reality, these hallmarks do not exist in isolation, and there is a large degree of crosstalk between signals which makes identification of targeted combinations challenging because of pathway redundancy. The potential network of interactions and crosstalk between targets is shown in Figure 2. Particularly, many of the signals which promote angiogenesis, proliferation and evading apoptosis can also act to enhance immune evasion and invasion and metastases. For example, activation of STAT3 (which can be inhibited through JAKi), can be initiated through EGFR or IL-6R. As a master regulator of transcription STAT3 can promote other TFs associated with EMT including Snail and ZEB1 leading to invasion. Additionally, STAT3 has been observed to increase production of PDL1 on tumour cells and can crosstalk with NF*κ*B to enhance proliferation and resistance to apoptosis in tumour cells. This complexity requires further investigation in the setting of RT and RC and should be assessed in cases where novel combinations fail to achieve good clinical effects in trials.

Advancements in our understanding of tumour heterogeneity have highlighted the need for a stratified/precision oncology to improve rectal cancer patient outcomes. Clinically relevant translatable predictive biomarkers for determining optimal therapy combinations for every patient remain a key area of ongoing research. This review has highlighted a variety of promising targeted therapy: radiation combinations which could provide clinical benefit for subsets of rectal cancer patients.
